# Folic Acid-Appended Hydroxypropyl-β-Cyclodextrin Exhibits Potent Antitumor Activity in Chronic Myeloid Leukemia Cells via Autophagic Cell Death

**DOI:** 10.3390/cancers13215413

**Published:** 2021-10-28

**Authors:** Toshimi Hoshiko, Yasushi Kubota, Risako Onodera, Taishi Higashi, Masako Yokoo, Keiichi Motoyama, Shinya Kimura

**Affiliations:** 1 Division of Hematology, Respiratory Medicine and Oncology, Department of Internal Medicine, Faculty of Medicine, Saga University, Saga 849-8501, Japan; 19624015@edu.cc.saga-u.ac.jp (T.H.); kubotay@saitama-med.ac.jp (Y.K.); 2 Saitama Medical Center, Department of Transfusion Medicine and Cell Therapy, Saitama Medical University, Kawagoe 350-8550, Japan; 3 Graduate School of Pharmaceutical Sciences, Kumamoto University, Kumamoto 862-0973, Japan; deragon@kumamoto-u.ac.jp (R.O.); higashit@kumamoto-u.ac.jp (T.H.); motoyama@kumamoto-u.ac.jp (K.M.); 4 Priority Organization for Innovation and Excellence, Kumamoto University, Kumamoto 862-0973, Japan; 5 Saga Medical Center Koseikan, Department of Hematology, Saga 849-8571, Japan; yokoo-m@koseikan.jp

**Keywords:** HP-β-CyD, folic acid, folate receptor, BCR-ABL, cholesterol, mitophagy, autophagy, autophagic cell death, tumor targeting

## Abstract

**Simple Summary:**

2-Hydroxypropyl-β-cyclodextrin (HP-β-CyD) is a cyclic oligosaccharide widely used as an excipient in pharmaceutical preparations, in addition to also being used as a cholesterol regulator. HP-β-CyD was used in clinical trials for patients with Niemann-Pick Type C disease to remove accumulated intracellular lipid. HP-β-CyD has anti-leukemia activity by inducing apoptosis and cell-cycle arrest; however, the antitumor activity of HP-β-CyD lacks tumor cell-selectivity. Taking advantage of the fact that folate receptors are highly expressed in many cancer cells, we synthesized folate-appended HP-β-CyD (FA-HP-β-CyD) to confer tumor cell-selectivity to HP-β-CyD. FA-HP-β-CyD inhibited the proliferation of chronic myeloid leukemia (CML) cells and the mechanism underlying the effect of FA-HP-β-CyD in inducing cell death may involve autophagy. The combination of FA-HP-β-CyD and ABL tyrosine kinase inhibitors (imatinib and ponatinib) had a synergistic inhibitory effect on CML cells. In a mouse model of BCR-ABL-induced leukemia, FA-HP-β-CyD had a stronger inhibitory effect on leukemia progression than HP-β-CyD or imatinib.

**Abstract:**

2-Hydroxypropyl-β-cyclodextrin (HP-β-CyD) is widely used as an enabling excipient in pharmaceutical formulations. We previously demonstrated that HP-β-CyD disrupted cholesterol homeostasis, and inhibited the proliferation of leukemia cells by inducing apoptosis and cell-cycle arrest. Recently developed drug delivery systems using folic acid (FA) and folic acid receptors (FR) are currently being used in cancer treatment. To confer tumor cell-selectivity to HP-β-CyD, we synthesized folate-appended HP-β-CyD (FA-HP-β-CyD) and evaluated the potential of FA-HP-β-CyD as an anticancer agent using chronic myeloid leukemia (CML) cells in vitro and in vivo. FA-HP-β-CyD inhibited the growth of FR-expressing cells but not that of FR-negative cells. FA-HP-β-CyD had stronger anti-leukemia and cell-binding activities than HP-β-CyD in CML cells. Unlike HP-β-CyD, FA-HP-β-CyD entered CML cells through endocytosis and induced both apoptosis and autophagy via mitophagy. FA-HP-β-CyD increased the inhibitory effects of the ABL tyrosine kinase inhibitors imatinib mesylate and ponatinib, which are commonly used in CML. In vivo experiments in a BCR-ABL leukemia mouse model showed that FA-HP-β-CyD was more effective than HP-β-CyD at a ten-fold lower dose. These results indicate that FA-HP-β-CyD may be a novel tumor-targeting agent for the treatment of leukemia.

## 1. Introduction

Chronic myeloid leukemia (CML) is caused by the development of the Philadelphia (Ph) chromosome; the resulting fusion gene encodes the BCR-ABL chimeric protein, which has stronger kinase activity than ABL [1]. The introduction of imatinib mesylate, an ABL tyrosine kinase inhibitor (TKI), followed by the development of second and third generation TKIs (dasatinib, nilotinib, bosutinib, and ponatinib), has dramatically improved the prognosis of patients with CML [2,3]. Although these drugs prolonged the life expectancy of patients with CML to a level similar to that of the general population, resistance and intolerance to TKIs still occur. Thus, the development of innovative therapies that promote cell death in neoplasms is desired.

2-Hydroxypropyl-β-cyclodextrin (HP-β-CyD) is used clinically as a pharmaceutical diluting agent for drugs with poor water solubility and as a cholesterol modifier. HP-β-CyD is registered for oral, buccal, rectal, ophthalmic, and intravenous use [4]. Oral and intravenous solutions containing HP-β-CyD in a complex with itraconazole, a broad-spectrum triazole antifungal agent, are widely used [5]. HP-β-CyD was used in clinical trials for patients with Niemann-Pick Type C disease to remove accumulated intracellular lipid [6,7]. Considering that cholesterol is essential for the proliferation and maintenance of tumor cells, we hypothesized that HP-β-CyD might have antitumor effects and tested its growth inhibitory effects on leukemia cells, including CML cells, in vitro and in vivo [8]. However, the cytotoxic activity of HP-β-CyD lacks tumor cell-selectivity.

Drug delivery systems using folic acid (FA) and folic acid receptors (FRs) were recently developed and are being applied for the diagnosis and treatment of cancer. FR is expressed at low levels in normal cells, whereas its expression is high in many cancer cells, except in A549 cells derived from lung cancer patients, among others [9,10]. These findings have stimulated research into FA-conjugated drugs, which are currently being developed for clinical application. FA-M-β-CyD, which couples FA with M-β-CyD, was recently developed and shown to possess cancer cell-killing capacity [11,12]. FA-M-β-CyD induces mitophagy by inhibiting the deposition of cholesterol on the mitochondrial membrane after internalization into the cell and induces cell death in a caspase-3/7-independent manner [13,14].

**Figure 1:**
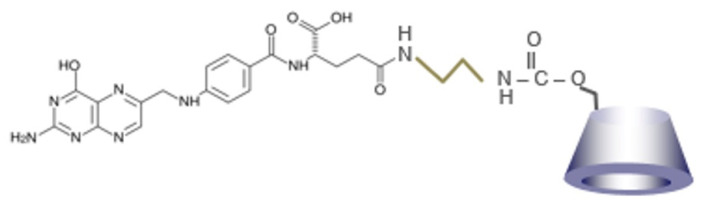
Chemical structure of FA-HP-β-CyD.

In the present study, we aimed to impart tumor cell-selective cytotoxicity to HP-β-CyD by synthesizing folate-appended HP-β-CyD (FA-HP-β-CyD, Figure 1). We investigated its effect against CML cells in vitro and in vivo, as well as the underlying mechanism.

## 2. Materials and Methods

### 2.1. Reagents and Cell Lines

HP-β-CyD was purchased from Tokyo Chemical Industry (Tokyo, Japan). The average molecular weight of HP-β-CyD is 1391. For in vitro use, the compound was dissolved in saline (Otsuka Pharmaceuticals, Tokyo, Japan) to a concentration of 500 mM. For in vivo use, HP-β-CyD was dissolved in saline to a concentration of 150 mM. The solutions were stored at 4 °C until use.

The human CML cell lines BV173 and K562 were purchased from DSMZ (Braunschweig, Germany) and the American Type Culture Collection (ATCC, Manassas, VA, USA), respectively. An imatinib-resistant Lyn-overexpressing CML cell line, specifically MYL-R, and the KBM-5/STI cell line were kindly provided by Dr. H. Tanaka (Hiroshima City Asa Hospital, Hiroshima, Japan) and Dr. M. Beran (University of Texas M.D. Anderson Cancer Center, Houston, TX, USA), respectively [15,16]. Ba/F3 cell lines expressing p190 or p210 Bcr-Abl/wild-type (WT) were established as described previously [17]. Cells were maintained as suspension cultures in RPMI-1640 (Sigma-Aldrich, St. Louis, MO, USA), supplemented with 10% heat-inactivated fetal calf serum (FCS; Nichirei Bioscience Inc., Tokyo, Japan) at 37 °C in a fully humidified chamber containing 5% CO_2_ in the air. The lung cancer cell line, specifically A549 cells, and human primary hepatocyte were purchased from JCRB Cell Bank (Ibaraki, Japan) and ATCC, respectively, and were maintained as suspension cultures in DMEM (Sigma-Aldrich) supplemented with 10% FCS.

### 2.2. Preparation of FA-HP-β-CyD

FA was conjugated with ethylenediamine (EDA) to introduce an amino group. FA (880 mg) was dissolved in 40 mL of anhydrous DMSO. Then, N, N’-dicyclohexylcarbodiimide (DCC, MW = 206, 412 mg) and N-hydroxysuccinimide (NHS, MW = 115, 230 mg) were added, and the mixture was stirred for 20 h. EDA (MW = 60.1, 1 mL) was added and the mixture was further stirred at room temperature for 24 h. FA-EDA was obtained by washing with acetonitrile three times, followed by suction filtration.

Next, HP-β-CyD was activated with 1,1′-carbonyldiimidazole (CDI) to synthesize CDI-activated HP-β-CyD. HP-β-CyD (MW = 1541, 1.5 g) was dissolved in 20 mL of DMSO. Triethylamine (MW = 101, 100 μL) and CDI (MW = 162, 1.62 g) were added, and the sample was stirred for 16 h at room temperature. Finally, FA-EDA was reacted with CDI-activated HP-β-CyD to obtain FA-HP-β-CyD. FA-EDA was added to CDI-activated HP-β-CyD and stirred for 72 h at room temperature. After dialysis in 0.1 mM of ammonia solution for 72 h (MWCO 1000), the samples were lyophilized, followed by suction filtration and evaporation. The preparation was confirmed by MALDI-TOF-MS and ^1^H-NMR. Figure 1 shows the chemical structure of FA-HP-β-CyD.

### 2.3. Mice

For in vivo transplantation experiments, Balb/cAJcl-nu/nu (nude) mice (5–6 weeks old, SLC Japan, Inc., Shizuoka, Japan) were used. Animal studies were conducted in accordance with the institutional guidelines of animal care of Saga University and with the approval of the Institutional Review Board of Saga University.

### 2.4. Analysis of Cell Growth and Viability

The trypan blue dye exclusion method was used to evaluate cell viability. A modified methyl-thiazolyl-diphenyl-tetrazolium (MTT) assay with the Cell Counting Kit-8 (Dojindo Molecular Technologies, Kumamoto, Japan) was used to evaluate cell proliferation, as previously described [18]. The non-linear regression program CalcuSyn (Biosoft, Cambridge, UK) was used to determine the values of 50% inhibitory concentration (IC_50_).

After obtaining IC_50_ values for each agent, the anti-leukemic activity of FA-HP-β-CyD combined with TKIs (imatinib and ponatinib) was evaluated, as previously described [19]. Five concentrations (0.25, 0.5, 0.75, 1.0, and 2.0-folds the IC_50_) of FA-HP-β-CyD and imatinib or ponatinib were used to treat K562 and BV173 cells. CalcuSyn was used to calculate the fraction affected (Fa, in which a value of 0.25 indicates 25% viable cells) and the combination index (CI). This method is capable of quantifying synergistic (CI < 1) and antagonistic (CI > 1) effects at different doses and effect levels.

### 2.5. In Vitro Antitumor Activity in the Presence of a Competitive Inhibitor of FR

K562 and BV173 cells (1 × 10^5^) were treated with FA-HP-β-CyD (5 mM), HP-β-CyD (5 mM), and FA-HP-β-CyD (5 mM) with folate (4 mM), HP-β-CyD (5 mM) with folate (4 mM), and folate alone (4 mM) at 37 °C for 2 h. Cell proliferation was assessed using the Cell Counting Kit-8. Data are presented as the mean ± standard deviation (SD) of three independent experiments.

### 2.6. Flow Cytometric Analysis

To detect the expression of FR, cell suspensions of 2 × 10^6^ leukemia cells were incubated with PE anti-FOLR1 antibody, APC anti-human FR-β antibody, and APC anti-mouse FR-β antibody (Bio Legend, San Diego, USA). Propidium iodide (PI) and Annexin V (BD Biosciences, San Jose, CA, USA)-staining was performed to assess apoptosis according to the manufacturer’s instructions. The cells were filtered through nylon mesh and analyzed using a FACSVerse flow cytometer (BD Biosciences).

### 2.7. Western Blot Analysis

Western blot analysis was performed by our laboratory protocol as previously described [20]. Antibodies against LC3B (Abcam, Cambridge, UK) and alpha tubulin (Proteintech, Rosemont, IL, USA) were used. For the secondary antibodies, IRDye^®^ 800CW goat anti-rabbit IgG and IRDye^®^ 680LT goat anti-mouse IgG (LI-COR Biosciences, Lincoln, NE, USA) were used. All original western blot figures are included in Figure S8.

### 2.8. Cellular Association of FA-HP-β-CyD

To investigate the cellular association of FA-HP-β-CyD, Tetramethylrhodamine isothiocyanate (TRITC)-labeled FA-HP-β-CyD (TRITC FA-HP-β-CyD) was used. For the preparation of TRITC FA-HP-β-CyD, 10 mg of FA-HP-β-CyD and 1 mg of TRITC were dissolved in 400 μL of DMSO. After stirring for 24 h, the solution was gradually poured into acetone. The precipitant was obtained and dissolved with water. After freeze drying, TRITC-FA-HP-β-CyD was obtained. K562 and BV173 cells (1 × 10^6^) were incubated with 10 µM og TRITC-FA-HP-β-CyD at 37 °C for 1 h. After washing, the samples were scraped with 1 mL of PBS (pH 7.4). Data were acquired with a FACSVerse (BD Biosciences).

### 2.9. Intracellular Distribution of TRITC-FA-HP-β-CyD

Cells (1 × 10^6^) were treated with TRITC-FA-HP-β-CyD (1 mM), TRITC-HP-β-CyD (10 mM), and TRITC-FA-HP-β-CyD (1 mM) with folate (4 mM) at 37 °C for 1 h. The medium was removed and PlasMem Bright Green (×200, 200 uL, Dojindo Molecular Technologies) was both added and incubated at 37°C for 5 min. After centrifugation onto glass slides using Cytospin™ (Thermo Fisher Scientific, Waltham, MA, USA), cells were washed gently with PBS containing 0.1% Tween 20 and incubated in PBS (pH 7.4) containing 4% paraformaldehyde for 10 min at room temperature. Then, the samples were incubated with PBS containing 0.1% Triton X-100 for 10 min and washed three times with PBS. To block the non-specific binding of antibodies, the samples were incubated with blocking solution for 30 min at room temperature and slides were sealed with mounting medium with DAPI. For cell observation, the confocal laser microscope LSM-880 (Carl Zeiss, Jena, German) was used. The results were analyzed by Imaris software (Carl Zeiss).

### 2.10. Activation of Caspases 3/7

Cells were incubated with FA-HP-β-CyD (1 mM and 5 mM) and HP-β-CyD (1 mM and 5 mM) for 48 h. After washing twice with 1 mL of RPMI-1640 medium (FA-free), cells were added to 10 mM of CellEvent™ Caspase-3/7 Green Detection Reagent (Invitrogen, Tokyo, Japan) and incubated at 37 °C for 30 min. The cells were washed twice and 1 mL of RPMI-1640 medium was added. The cells were observed under the fluorescence microscope AxioImager.M2 (Carl Zeiss Microscopy, Jena, Germany). The results were analyzed by Imaris software.

### 2.11. Autophagosome Formation

CML cell lines (K562 and BV173) were cultured in 6-well plates at a density of 4 × 10^5^ cells/well and treated with 1 mM or 5 mM of HP-β-CyD or FA-HP-β-CyD for 2 h. Subsequent processing was performed as described in “2.9. Intracellular distribution of TRITC-FA-HP-β-CyD”. After incubation with the blocking solution for 30 min at room temperature, samples were incubated with anti-LC3B (Abcam) for 1 h at room temperature or overnight at 4 °C in a wet box. Then, the samples were incubated with fluorescently labeled secondary antibody goat anti-rabbit IgG H&L (Abcam) for 1 h at room temperature in the dark and sealed with mounting medium with DAPI. The cells were observed using the fluorescence microscope AxioImager.M2. The results were analyzed by Imaris software.

### 2.12. Cell Culture Using Autophagy Inhibitors

CML cell lines (2 × 10^5^ cells in 100 μL of RPMI-1640 medium per well) were seeded in flat-bottomed 96-well plates (Greiner Labortechnik, Hamburg, Germany) and treated with both 5 mM of FA-HP-β-CyD and 15 mM of HP-β-CyD at 37 °C for 2 h. The cells were then pretreated with the autophagy inhibitors chloroquine (20 µM), LY294002 (50 μM) (Fujifilm Wako Pure Chemicals Co., Ltd.), or bafilomycin A1 (1 nM) (Funakoshi, Tokyo, Japan) for 24 h. Cell proliferation was assessed using the Cell Counting Kit-8. Data are presented as the mean ± SD of three independent experiments.

### 2.13. Detection of Mitophagy

Mitophagy was detected in K562 and BV173 cells using the Mitophagy detection kit^®^ (Dojindo Molecular Technologies) containing Mtphagy Dye^®^ and Lyso Dye^®^. Cells were treated with 100 nM of Mtphagy dye for 15 min. After washing with medium, the cells were treated with 10 mM of HP-β-CyD and 1 mM of FA-HP-β-CyD for 2 h. After washing with medium, 1 µM of Lyso dye was added and further incubated for 10 min. After washing with Hanks’ balanced salt solution, the cells were observed under the fluorescence microscope AxioImager.M2 and the results were analyzed by Imaris software.

### 2.14. Intracellular Adenosine Triphosphate (ATP) Quantification

The IntraCellular ATP assay kit (Cosmo Bio, Tokyo, Japan) was used to quantify intracellular ATP levels according to the manufacturer’s instructions. K562 and BV173 cells (2 × 10^4^/well) were cultured in 96-well culture plates. For the assay, cells were washed with RPMI medium and incubated with both 10 mM of HP-β-CyD and 1 mM of FA-HP-β-CyD at 37 °C for 2 h. Then, cells were incubated with 100 µL of ATP determination reagent for 10 min. A fluorescence microplate reader (Varioskan Flash; Thermo Fisher Scientific) was used to quantify the fluorescence derived from the ATP production.

### 2.15. ROS Generation Assay

Intracellular ROS generation was detected using ROS-ID^®^ (Enzo Life Sciences). Cells (2 × 10^5^/well) were incubated with 1 mM of FA-HP-β-CyD and 10 mM of HP-β-CyD at 37 °C for 2 h. Then, 1 mL of total ROS detection reagent in medium was added. Data were obtained on a FACSVerse (BD Biosciences).

### 2.16. Statistical Analysis

Data are given as the mean ± SD. The unpaired two-tailed Student’s *t*-test was used to analyze the data; values of *p* < 0.05 were considered significant. To evaluate in vivo efficacy, survival curves were generated using the Kaplan–Meier method and compared by the log-rank test.

## 3. Results

### 3.1. Leukemia Cell Lines Highly Express Folate Receptor β but Not Folate Receptor α

**Figure 2:**
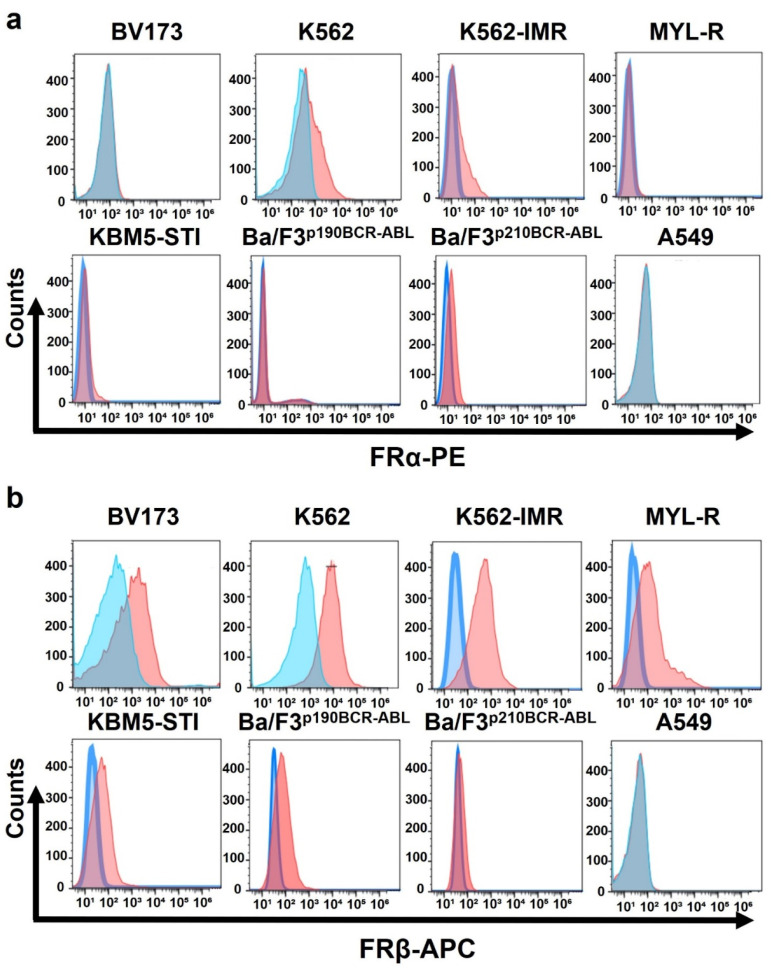
Expression of folate receptors α and β in CML cell lines. (**a**) Folate receptor α (FRα) expression in CML cells was measured by flow cytometry using FRα-PE antibody. (**b**) Folate receptor β (FRβ) expression in CML cells was measured by flow cytometry using FRβ-APC antibody. Blue: control; orange: stained sample with anti-FRα or FRβ antibody.

First, we investigated the expression of FR in CML cell lines using flow cytometry (Figure 2). FRβ was strongly expressed in the leukemia cells examined (Figure 2b), whereas FRα expression was weak or negative (Figure 2a). FR was not expressed in A549 cells or hepatocytes (Figure 2a,b, and Figure S1).

### 3.2. Anti-Leukemia Effect of FA-HP-β-CyD

**Figure 3:**
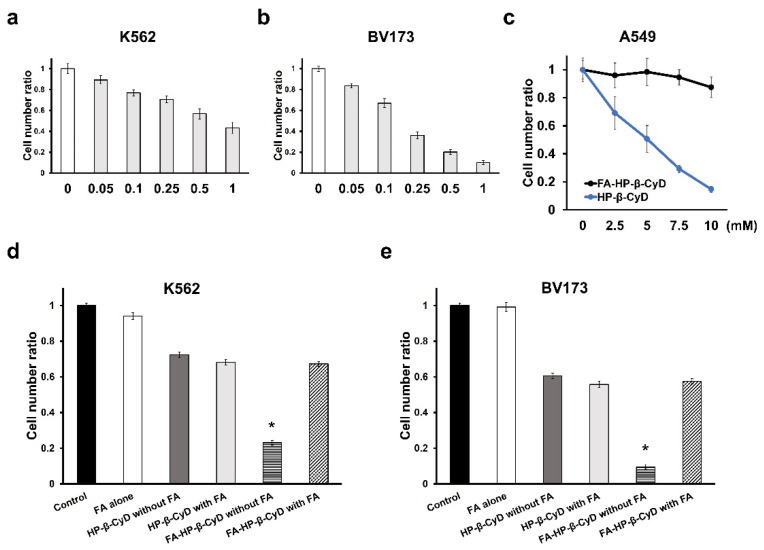
Antitumor effect of FA-HP-β-CyD. (**a**,**b**) Antitumor activity of FA-HP-β-CyD (0, 0.05, 0.1, 0.25, 0.5, and 1.0 mM) in K562 and BV173 cells. Cells were incubated with FA-HP-β-CyD for 72 h at 37 °C. (**c**) Antitumor activity of FA-HP-β-CyD and HP-β-CyD in A549 cells. Cells were incubated with 0, 2.5, 5, 7.5, and 10 mM of FA-HP-β-CyD or HP-β-CyD for 72 h at 37 °C. (**d**,**e**) Treatment with FA-HP-β-CyD in the presence or absence of FA. K562 and BV173 cells were incubated for 2 h at 37 °C with medium only (control), medium containing FA-HP-β-CyD (5 mM) and HP-β-CyD (5 mM) in the absence and presence of FA (2 mM). Each value represents the mean ± SEM of three experiments. * *p* < 0.05. The cell number ratio is the number of cells calculated with the number of cells in the control as 1.

We investigated whether FA-HP-β-CyD has FR-positive cell-selective anti-leukemia effects. FA-HP-β-CyD showed significant anti-leukemia activity in both K562 and BV173 cells (Figure 3a,b). HP-β-CyD displayed potent cytotoxicity against A549 cells with negative FR expression, whereas FA-HP-β-CyD had no cytotoxic effect (Figure 3c). These results suggest that the anti-leukemia activity of FA-HP-β-CyD was selective against FR-expressing cells. Next, we evaluated the effect of FA, a competitor of FR, on the anti-leukemia activity of β-CyDs in CML cell lines. The anti-leukemia activity of FA-HP-β-CyD, but not that of HP-β-CyD, was significantly inhibited by the addition of FA (Figure 3d,e). These data indicated that FA-HP-β-CyD had FR-mediated anti-leukemia activity.

### 3.3. FA-HP-β-CyD Inhibits the Growth of Leukemia Cell Lines More Potently than HP-β-CyD

We evaluated the effect of FA-HP-β-CyD on the viability of leukemia cells. IC_50_ values of FA-HP-β-CyD were determined in seven CML cell lines, including imatinib-resistant cell lines (K562-IMR, MYL-R, and KBM-STI), and after 72 h of exposure, they ranged from 0.17 to 0.96 mM (Table 1). The IC_50_ values of FA-HP-β-CyD were at least five-folds lower than those of HP-β-CyD.

### 3.4. FA-HP-β-CyD Inhibits Cell Growth by Inducing Apoptosis

**Figure 4:**
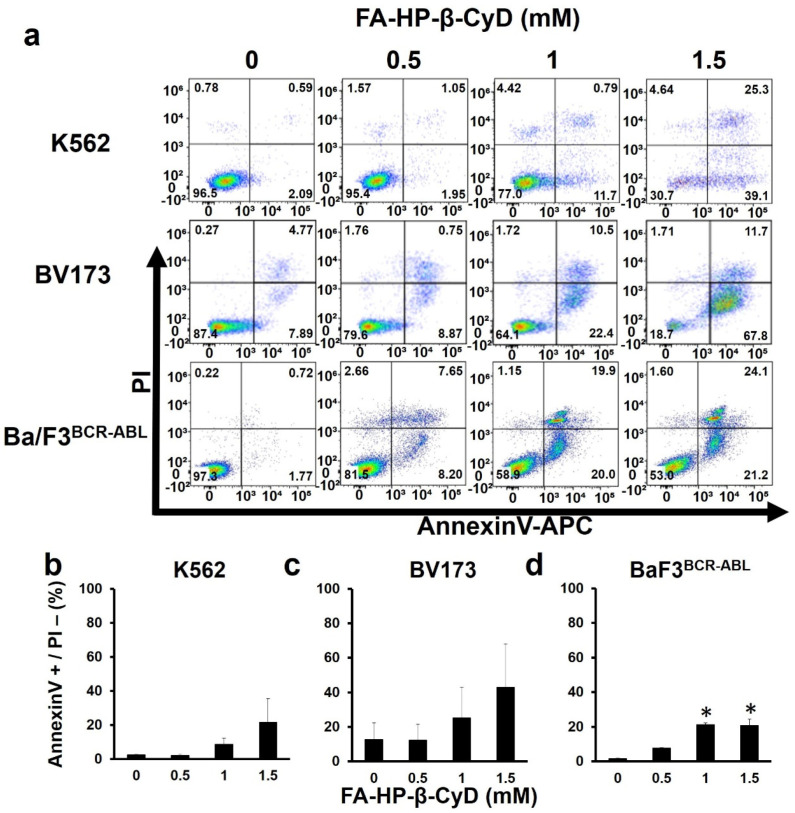
FA-HP-β-CyD induces apoptosis in K562 cells, BV173 cells, and Ba/F3^BCR-ABL^ cells. (**a**) K562 cells, BV173 cells, and Ba/F3^BCR-ABL^ cells were treated with 0, 0.5, 1.0, and 1.5 mM of FA-HP-β-CyD. After 72 h of culture, the Annexin V and PI-staining was done. Representative FACS plots are shown (*n* = 3). (**b**–**d**) Percentages of Annexin V-positive PI-negative cells exposed to FA-HP-β-CyD for 72 h are shown. Data represent the mean ± SD of three independent experiments. * *p* < 0.05.

Annexin V and PI-staining was performed to evaluate the apoptosis of FA-HP-β-CyD-treated leukemia cells. Exposure to FA-HP-β-CyD resulted in apoptosis in both BV173 and K562 cells in a dose-dependent manner (Figure 4a–c). K562 cells exposed to 1.5 mM of FA-HP-β-CyD seemed to have gone into necrosis. Similar results were obtained in Ba/F3^BCR-ABL^ cells treated with FA-HP-β-CyD (Figure 4a,d). Caspase 3/7 activity was investigated in K562 and BV173 cells treated with CyDs (1 and 5 mM) using the CellEvent Caspase 3/7 detection reagent. We previously reported that HP-β-CyD at these concentrations causes almost no apoptosis [8]. Treatment of both cell lines with 1 and 5 mM of FA-HP-β-CyD, but not HP-β-CyD, for 2 h caused caspase 3/7 activation (Figure S2).

### 3.5. Cellular Uptake of FA-HP-β-CyD

**Figure 5:**
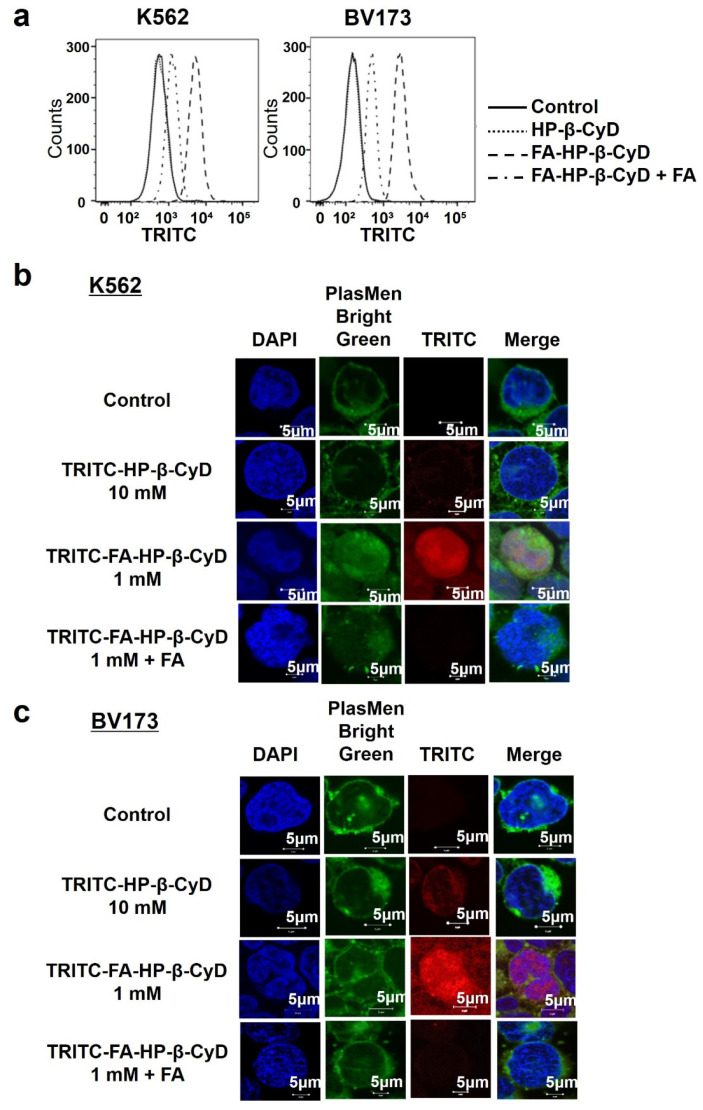
Intracellular distribution of FA-HP-β-CyD. (**a**) Cellular association of TRITC-FA-HP-β-Cy in K562 cells (left) and BV173 cells (right). The fluorescence intensity derived from TRITC was determined by flow cytometry at 1 h after incubation at 37 °C. Control: medium only. (**b**,**c**) Intracellular distribution of TRITC-FA-HP-β-CyD. CML cells were treated with medium only (control), TRITC-FA-HP-β-CyD (1 mM), TRITC-FA-HP-β-CyD (1 mM) and FA (2 mM), or TRITC-HP-β-CyD (10 mM) for 1 h. The experiments were performed three times independently and representative images are shown.

CyD uptake into cells is difficult because of its hydrophilic nature and high molecular weight of approximately 1000 [21]. However, FA-HP-β-CyD possessed excellent selective antitumor activity against cells expressing high levels of FRβ, suggesting that its antitumor activity is involved in FR-mediated cellular uptake. To examine the mechanism underlying the cytotoxic effect of FA-HP-β-CyD, we examined whether TRITC-FA-HP-β-CyD associates with CML cell lines. TRITC-FA-HP-β-CyD associated with both K562 and BV173 cells (Figure 5a), even though CyDs do not permeabilize the biomembrane. Furthermore, the association of TRITC-FA-HP-β-CyD was inhibited when FA was added as a competitor of FR (Figure 5a), indicating that FA-HP-β-CyD associated with cells via FR-β.

Next, we examined the intracellular distribution of TRITC-FA-HP-β-CyD in CML cell lines after 1 h of treatment. The intracellular distribution of TRITC-HP-β-CyD was almost negligible; however, TRITC-FA-HP-β-CyD was taken up by CML cells and its uptake was suppressed by the addition of FA (Figure 5b,c).

### 3.6. FA-HP-β-CyD Induces Autophagosome Formation

**Figure 6:**
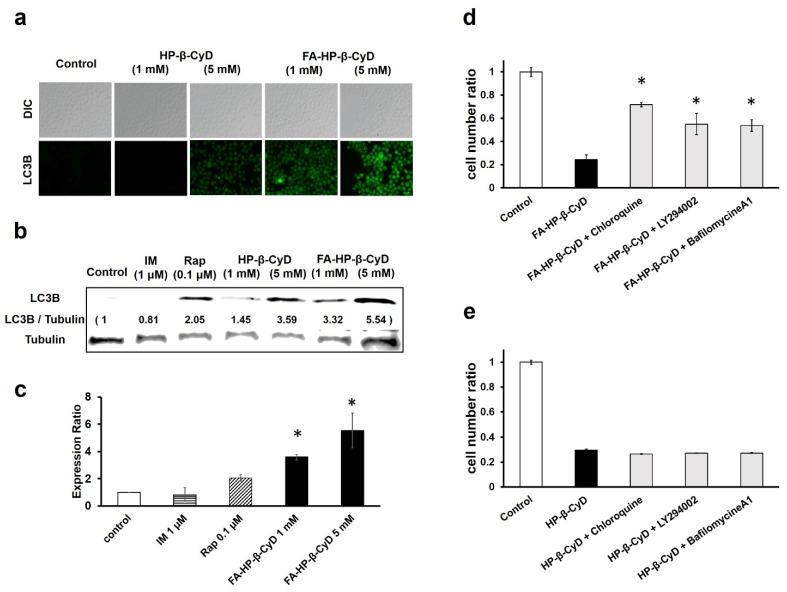
Induction of autophagy by treatment with FA-M-β-CyD. (**a**) Detection of autophagy in CML cells. K562 cells treated with FA-HP-β-CyD and HP-β-CyD for 2 h were exposed to Cyto-ID for 30 min. For observation, fluorescence microscopy was used. (**b**) Effect of HP-β-CyDs on LC3B expression in K562 cells. Cells were treated with medium only (control), FA-HP-β-CyD, HP-β-CyD, imatinib (IM), and rapamycin (Rap) for 2 h. LC3B protein levels were detected by western blotting. (**c**) The graph shows the fluorescence intensity of the bands. * *p* < 0.05 compared with the control. (**d**,**e**) Effects of chloroquine, bafilomycin A1, and LY294002 on the antitumor activity of FA-HP-β-CyD (**d**) and HP-β-CyD (**e**) in BV173 cells. Cells were incubated for 24 h. * *p* < 0.05 compared with FA-HP-β-CyD.

The effect of FA-HP-β-CyD in inducing apoptosis does not explain its ten-fold stronger effect, compared to that of HP-β-CyD, in inhibiting cell proliferation. Experiments using KB cells suggest that the antitumor activity of FA-M-β-CyD is mediated by autophagy [13]. Thus, the effect of FA-HP-β-CyD on autophagosome formation was investigated. For this purpose, we measured LC3B-derived fluorescence in response to FA-HP-β-CyD treatment (Figure 6a and Figure S3a) and the expression of the LC3B protein was confirmed by western blotting (Figure 6b and Figure S3b). The addition of LY294002, an autophagy inhibitor, markedly decreased LC3B-derived fluorescence and LC3B protein expression (Figure S4). These results suggest that FA-HP-β-CyD induces autophagy in CML cells.

### 3.7. The Antitumor Activity of FA-HP-β-CyD Is Reduced by Autophagy Inhibition

Considering that FA-HP-β-CyD selectively induced autophagosome formation in cells expressing high levels of FR, we examined the role of autophagy in the antitumor activity of FA-HP-β-CyD by treating CML cells with autophagy inhibitors, including bafilomycin A1, chloroquine, and LY294002, and examining cell viability after treatment with FA-HP-β-CyD. Bafilomycin A1 and chloroquine prevented endosomal acidification, thereby inhibiting both autophagosome and lysosome fusion, as well as lysosomal proteolysis. LY294002 was used as a PI3K inhibitor. As shown in Figure 6d and Figure S3d, CML cell viability was higher in cells treated with FA-HP-β-CyD in the presence of autophagy inhibitors than in those treated with FA-HP-β-CyD alone. The survival of cells treated with HP-β-CyD was not affected by the presence or absence of autophagy inhibitors (Figure 6e and Figure S3e). These data indicate that FA-HP-β-CyD may cause autophagic cell death in CML cells.

### 3.8. Induction of Mitophagy and Mitochondrial Dysfunction by FA-HP-β-CyD

**Figure 7:**
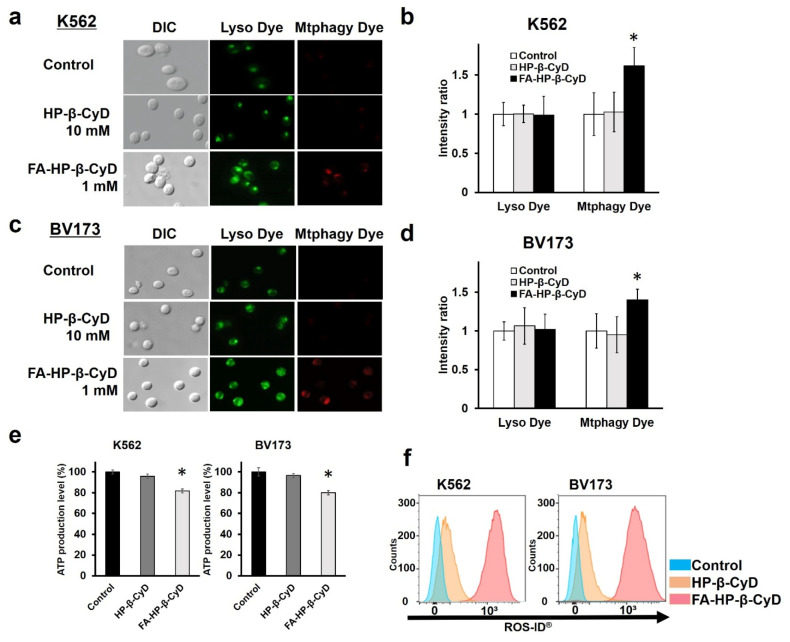
Detection of mitophagy, measurement of intracellular ATP levels, and ROS generation assay in CML cells. (**a**,**c**) Detection of mitophagy in K562 and BV173 cells. The cells were treated with Mtphagy dye, a mitophagy detection reagent, and then incubated with medium only (control), 1 mM of FA-HP-β-CyD, and 10 mM of HP-β-CyD, followed by Lyso dye, a lysosome detection reagent. Representative images are shown (*n* = 3). (**b**,**d**) Fluorescence intensities of Mtphagy dye and Lyso dye in the control, in HP-β-CyD-treated, and in FA-HP-β-CyD-treated cells are shown in bar graphs. * *p* < 0.05 compared with the control. (**e**) Measurement of intracellular ATP levels. K562 and BV173 cells were incubated with medium only (control), 1 mM of FA-HP-β-CyD, and 10 mM of HP-β-CyD for 2 h. Then, the cells were treated with ATP detection reagent. Bar graphs represent the mean ± SEM (*n* = 3 per group). * Significant difference with *p* < 0.05 compared with the control and FA-HP-β-CyD. (**f**) ROS generation assay. K562 and BV173 cells were incubated with medium only (control), 1 mM of FA-HP-β-CyD, and 10 mM of HP-β-CyD, and detected by flow cytometry using ROS detection reagents. Blue: control; orange: HP-β-CyD; and red: FA-HP-β-CyD.

Kameyama et al. reported that mitophagy induced by mitochondrial dysfunction may be associated with FA-M-β-CyD-induced autophagic cell death [14]. Thus, we examined whether FA-HP-β-CyD elicits mitophagy in CML cells. As shown in Figure 7a–d, mitophagic vacuoles and lysosomes stained by Mtphagy dye and Lyso dye, respectively, partially co-localized in CML cells treated with FA-HP-β-CyD but not in those treated with HP-β-CyD. Mtphagy dye-stained cells were not detected in HP-β-CyD-treated cells.

As mitochondria produce ATP, we examined the effect of FA-HP-β-CyD on ATP production in CML cells. FA-HP-β-CyD significantly inhibited ATP production in CML cells compared with that in the control and HP-β-CyD-treated cells (Figure 7e). However, in A549 cells, FA-HP-β-CyD had no effect on ATP production (Figure S5a). These results suggest that FA-HP-β-CyD inhibited ATP production in leukemia cells expressing FR but not in FR-negative cells.

Mitochondria are an important source of ROS and ROS promote autophagy [22,23]. We therefore investigated the effect of FA-HP-β-CyD on ROS production in CML cells. As shown in Figure 7f, FA-HP-β-CyD significantly increased ROS production compared with HP-β-CyD, indicating that FA-HP-β-CyD might enhance FR-mediated ROS production in CML cells. FA-HP-β-CyD had no effect on ROS production in A549 cells (Figure S5b).

### 3.9. FA-HP-β-CyD Synergistically Increases the Effect of ABL Tyrosine Kinase Inhibitors

**Figure 8:**
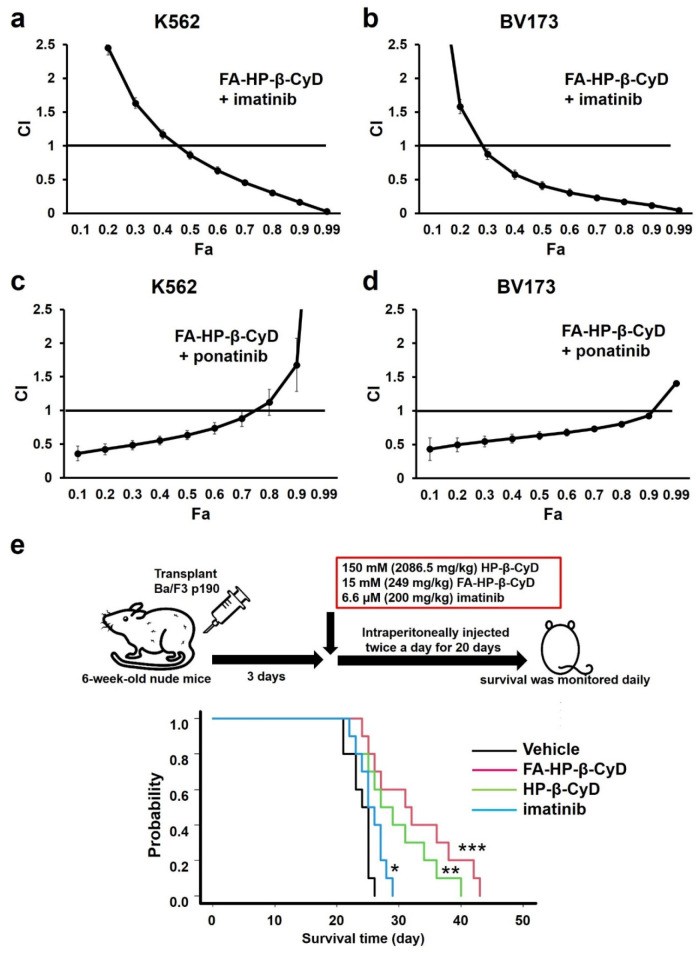
Evaluation of the combined effect of FA-HP-β-CyD and TKIs on CML cell lines, and effect of FA-HP-β-CyD on survival in a leukemia mouse model. (**a**–**d**) FA-HP-β-CyD was combined with imatinib mesylate or ponatinib. The combination index (CI) was calculated using Calcusyn and plotted as a function of the fraction affected (Fa). For example, 50% growth inhibition would result in a Fa of 0.5. We analyzed the synergistic (CI < 1), additive (CI = 1), or antagonistic (CI > 1) effects of combining multiple equal concentrations of drugs. The mean ± SD of three independent experiments is shown. (**e**) Survival curves of mice transplanted with Ba/F3 ^BCR-ABL^ cells positive for EGFP. Ba/F3 ^BCR-ABL^ cells (1 × 10^6^) were injected into nude mice. Three days after injection, 200 μL of the vehicle, 150 mM (2086.5 mg/kg) of HP-β-CyD, and 15 mM (249 mg/kg) of FA-HP-β-CyD were intraperitoneally administered twice a day. Imatinib mesylate was dissolved in deionized distilled water and 6.6 μM (200 mg/kg) of imatinib was given by oral gavage twice a day. The administration was continued for 20 days and survival was checked daily. Black lines, green lines, red lines, and blue lines indicate the survival curves of mice treated with the vehicle, with 150 mM of HP-β-CyD, 15 mM of FA-HP-β-CyD, and 6.6 μM of imatinib, respectively. Survival data were analyzed using a log-rank non-parametric test and are shown as Kaplan–Meier survival curves (*n* = 10). * *p* < 0.05, ** *p* < 0.01, and *** *p* < 0.001.

We investigated the effect of combination treatment with FA-HP-β-CyD and ABL TKIs (imatinib and ponatinib) on K562 and BV173 cells. A modified MTT assay was performed using five concentrations (0.25, 0.5, 0.75, 1.0, and 2.0-folds higher than the IC_50_) of each agent or a combination of the two at a constant ratio. The IC_50_ values obtained from the experiments above were used (Figure S6). The CI and Fa values for each dilution were calculated using CalcuSyn software, as reported previously [19]. Dose-effect and CI-Fa plots illustrating the effects of combinations of FA-HP-β-CyD and TKIs are presented in Figure 8. Combination treatment with FA-HP-β-CyD and imatinib or ponatinib had a stronger growth inhibitory effect than either agent alone. Mathematical analyses of the data presented in the CI-Fa plots are shown in Figure 8a–d. The CI values at Fa 0.5 of the combination of FA-HP-β-CyD with imatinib or ponatinib were 0.86 and 0.63, respectively, in K562 cells, and 0.41 and 0.63, respectively, in BV173 cells. These observations indicate that FA-HP-β-CyD had synergistic effects with imatinib or ponatinib on K562 and BV173 cells. We examined the effect of combination treatment with HP-β-CyD and imatinib, which showed that these agents acted antagonistically in BV173 cells, as indicated by CI values consistently exceeding 1.0 ± 1 SD for all fractions (Figure S7).

### 3.10. Administration of FA-HP-β-CyD Prolongs Survival in a Leukemia Mouse Model

The in vitro data demonstrated that FA-HP-β-CyD markedly inhibited the growth of leukemia cells. To investigate the antitumor activity of FA-HP-β-CyD in vivo, we generated a mouse model of BCR-ABL-induced leukemia and treated mice with FA-HP-β-CyD (Figure 8e). Ba/F3 BCR-ABL^WT^ cells were transplanted into nude mice. Starting 3 days after transplantation, mice were administered with 200 μL of the vehicle, 15 mM of FA-HP-β-CyD (249 mg/kg intraperitoneally (i.p.); b.i.d.), 150 mM of HP-β-CyD (2086.5 mg/kg (i.p.); b.i.d.), or imatinib mesylate (200 mg/kg) for 20 days. Flow cytometry was used to detect EGFP^+^ cells in the bone marrow of control mice to confirm Ba/F3 BCR-ABL^WT^ cell engraftment. All mice in the vehicle group died within 28 days. FA-HP-β-CyD-treated mice had a significantly longer overall survival than vehicle-injected mice (Figure 8e). The survival time of FA-HP-β-CyD-injected mice was statistically longer than that of imatinib-treated mice in the same experimental setting (*p* = 0.007).

## 4. Discussion

In the present study, we showed that FA-HP-β-CyD inhibited the proliferation of CML cells and exhibited FR-expressing cell-selective anti-leukemia activity. The mechanism underlying the effect of FA-HP-β-CyD in inducing cell death may involve autophagy. The combination of FA-HP-β-CyD and TKIs (imatinib and ponatinib), commonly used for the treatment of patients with CML, had a synergistic inhibitory effect on CML cells. In a mouse model of BCR-ABL-induced leukemia, FA-HP-β-CyD had a stronger inhibitory effect on leukemia progression than HP-β-CyD or imatinib.

We previously showed that HP-β-CyD disrupted cholesterol homeostasis and inhibited the proliferation of leukemia cells by inducing apoptosis and cell-cycle arrest [8]. Similar to M-β-CyD, HP-β-CyD is not easily taken up by cells; however, it causes apoptosis by inhibiting the function of cholesterol on cell membranes, such as lipid rafts. The addition of FA to HP-β-CyD increased the anti-leukemia effect of HP-β-CyD by at least five-folds in vitro, suggesting that the underlying mechanism was not only apoptosis but also other modes of cell death. Considering that FA-M-β-CyD induces autophagy [13,14], we examined the potential involvement of autophagy and mitophagy in the anti-leukemia effect of FA-HP-β-CyD. The results showed that FA-HP-β-CyD entered cells and induced not only apoptosis but also mitophagy, leading to autophagic cell death.

Autophagy is involved in drug resistance, survival, and growth in the tumor environment in various solid tumors and hematological malignancies, and the regulation of autophagy has attracted attention as a promising target for cancer therapy [24,25,26]. Knockout of Atg3, a gene essential for autophagy, in a CML mouse model markedly delays the onset of CML, indicating that autophagy underlies the pathogenesis of CML [27]. Treatment of human AML and CML cells with daunorubicin or cytarabine activates autophagy, and the inhibition of autophagy enhances the cell-killing effects of daunorubicin and cytarabine in vitro [28,29].

In human CML cell lines and patient samples, chloroquine selectively increases the sensitivity of leukemia cells to histone deacetylase inhibitors [30]. However, autophagic activity is necessary for the sensitivity to certain drugs. Autophagy is induced by treatment with retinoic acid or arsenic trioxide (ATO) in acute promyelocytic leukemia, and autophagy is essential for the degradation of PML-RARα by retinoic acid or ATO [31]. Autophagy is also essential for ATO-induced degradation of BCR-ABL in CML and the inhibitory effect of ATO on colony formation is attenuated by lysosome inhibition [32]. Thus, the role of autophagy in CML remains controversial. Further research is also needed to determine the role of autophagy in the anticancer effects of different agents. Several studies suggest that autophagy plays an important role in the maintenance of leukemia stem cells and in the resistance to TKIs in the stem cell fraction in CML in vitro [33,34,35]. However, the role of autophagy may be altered by environmental factors and it is not clear whether autophagy is also important for the maintenance of CML stem cells in vivo [36,37]. We plan to investigate the effect of FA-HP-β-CyD on CML stem cells in vitro and in vivo in the near future.

FA-HP-β-CyD had a synergistic effect with TKIs (imatinib and ponatinib) in K562 and BV173 cells. Yan et al. reported that M-β-CyD induces autophagic cell death and that the combination of M-β-CyD and imatinib increases cell death in K562 cells [38]; however, in the present study, HP-β-CyD alone did not induce autophagic cell death in K562 cells and both HP-β-CyD and imatinib had antagonistic effects when used in combination. Although the exact underlying mechanism remains unknown and needs to be verified in the future, we speculate that HP-β-CyD destroys the signaling platform for tyrosine kinase receptors by depleting cholesterol from lipid rafts in the plasma membrane. Another possibility is that FA-HP-β-CyD enters the cell and affects the function of mitochondria without excessively inhibiting the function of lipid rafts, thereby preserving the signaling pathway activated by BCR-ABL.

The meningeal involvement of leukemia/lymphoma is a major complication that is difficult to treat [39]. Intrathecal and systemic chemotherapies, which are the main therapeutic approaches, have limited efficacy and repetitive chemotherapy has severe side effects, including pancytopenia. Intracerebroventricular administration of HP-β-CyD is used for the treatment of Niemann-Pick Type C disease [40,41]. As FA-HP-β-CyD showed FR-expressing cell-selective antitumor activity, intrathecal administration of FA-HP-β-CyD may be an effective treatment for leukemic/lymphomatous meningitis with few side effects.

## 5. Conclusions

In summary, we evaluated the potential of FA-HP-β-CyD as a novel anti-leukemia agent in vitro and in vivo. FA-HP-β-CyD displayed potent in vitro anti-leukemia activity compared with HP-β-CyD in CML cells expressing FR, but not in A549 cells or in FR-negative cells. The selective antitumor activity of FA-HP-β-CyD against FR-expressing cells may be mediated by autophagy. FA-HP-β-CyD significantly prolonged survival in a mouse model of BCR-ABL leukemia compared to HP-β-CyD and imatinib. Further research is necessary to confirm the effect of FA-HP-β-CyD on CML stem cells and its potential for clinical application.

## Figures and Tables

**Table 1:** IC_50_ values of HP-β-CyDs in various CML cell lines.

IC_50_ of Cell Lines	IC_50_ (mM)	
Cell Line	HP-β-CyD	FA-HP-β-CyD
K562	5.65 ± 0.96	0.69 ± 0.19
BV173	3.46 ± 0.33	0.17 ± 0.06
Ba/F3p190 ^BCR-ABL^	8.04 ± 0.71	0.91 ± 0.16
Ba/F3p210 ^BCR-ABL^	9.21 ± 0.05	0.96 ± 0.02
MYL-R	8.17 ± 0.15	0.93 ± 0.11
K562-IMR	3.51 ± 0.12	0.70 ± 0.06
KBM5-STI	6.64 ± 0.18	0.80 ± 0.12
Hepatocyte	12.91 ± 2.73	20.10 ± 4.50

Values represent the mean ± SD of at least three independent experiments.

## Data Availability

The data presented in this study are available in the present article (and Supplementary Material).

## Supplementary Materials

The following are available online at https://www.mdpi.com/article/10.3390/cancers13215413/s1, Figure S1: Expression of folate receptor α and β in hepatocytes, Figure S2: Effect of HP-β-CyDs on caspase 3/7 activity in CML cell lines, Figure S3: Induction of autophagy by FA-M-β-CyD, Figure S4: Effect of an autophagy inhibitor on FA-HP-β-CyD, Figure S5: Measurement of intracellular ATP levels and ROS generation in A549 cells, Figure S6: Antitumor activity of imatinib and ponatinib, Figure S7: Evaluation of the combined effect of HP-β-CyD and imatinib on BV173 cells, Figure S8: Original western blot figures.
