# Supplementary material for: Folic Acid-Appended Hydroxypropyl-β-Cyclodextrin Exhibits Potent Antitumor Activity in Chronic Myeloid Leukemia Cells via Autophagic Cell Death
Source: Cancers (Basel). 2021 Oct 28;13(21):5413. doi: 10.3390/cancers13215413 (PMC8582559; doi:10.3390/cancers13215413)
Supplement: Supplementary file 1 [file cancers-13-05413-s001.zip › cancers-1415308-supplementary.pdf]

Supplementary material

Folic Acid-Appended Hydroxypropyl-β-Cyclodextrin Exhibits Potent Antitumor Activity in Chronic Myeloid Leukemia Cells via Autophagic Cell Death

Toshimi Hoshiko, Yasushi Kubota, Risako Onodera, Taishi Higashi, Masako Yokoo, Keiichi Motoyama and Shinya Kimura

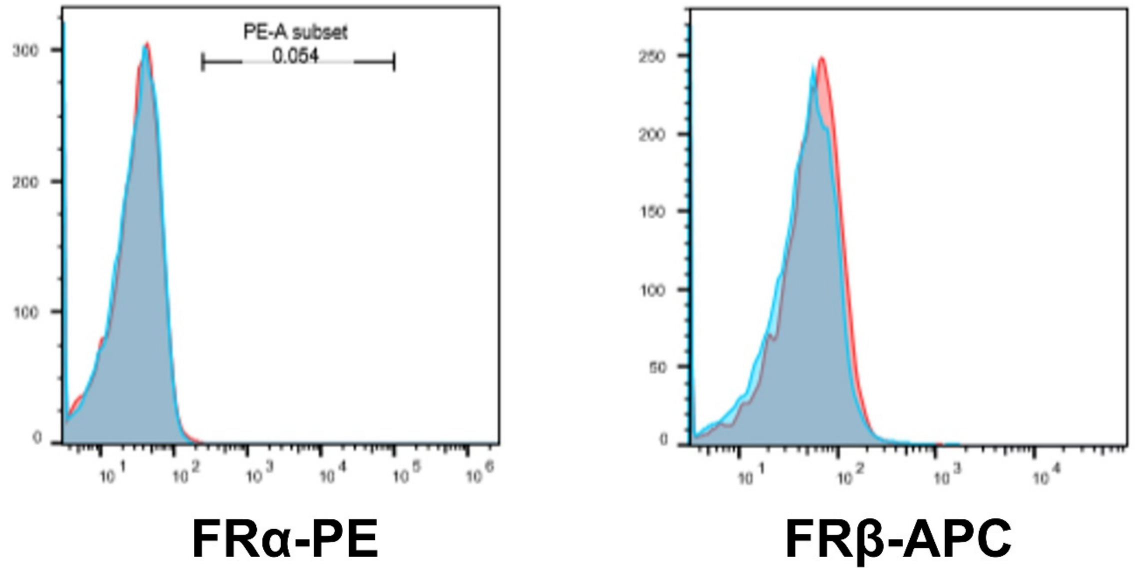

Figure S1. Expression of folate receptor α and β in hepatocytes. (a) Folate receptor α (FRα) in hepatocytes was measured by flow cytometry using FRα-PE antibody. (b) Folate receptor β (FRβ) in hepatocytes was measured by flow cytometry using FRβ-APC antibody.

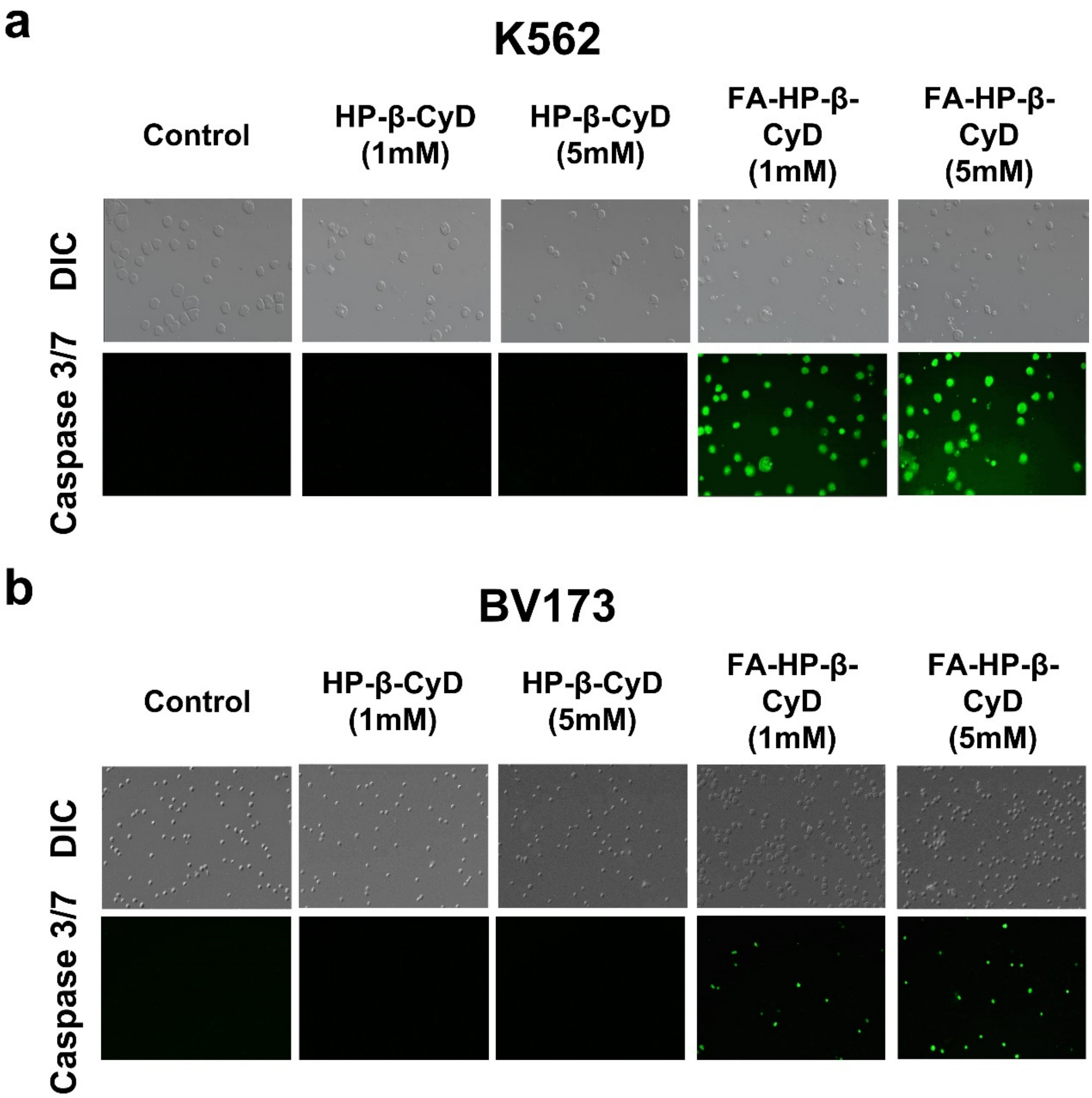

Figure S2. Effect of HP-β-CyDs on caspase 3/7 activity in CML cell lines. (a and b) K562 cells (a) and BV173 cells (b) were incubated with HP-β-CyD (1 mM, 5 mM) and FA-HP-β-CyD (1 mM, 5 mM) for 48 h, and 10 mM CellEvent™ Caspase-3/7 Green Detection Reagent was added and incubated at 37°C for 30 min. The cells were observed with a fluorescence microscope.

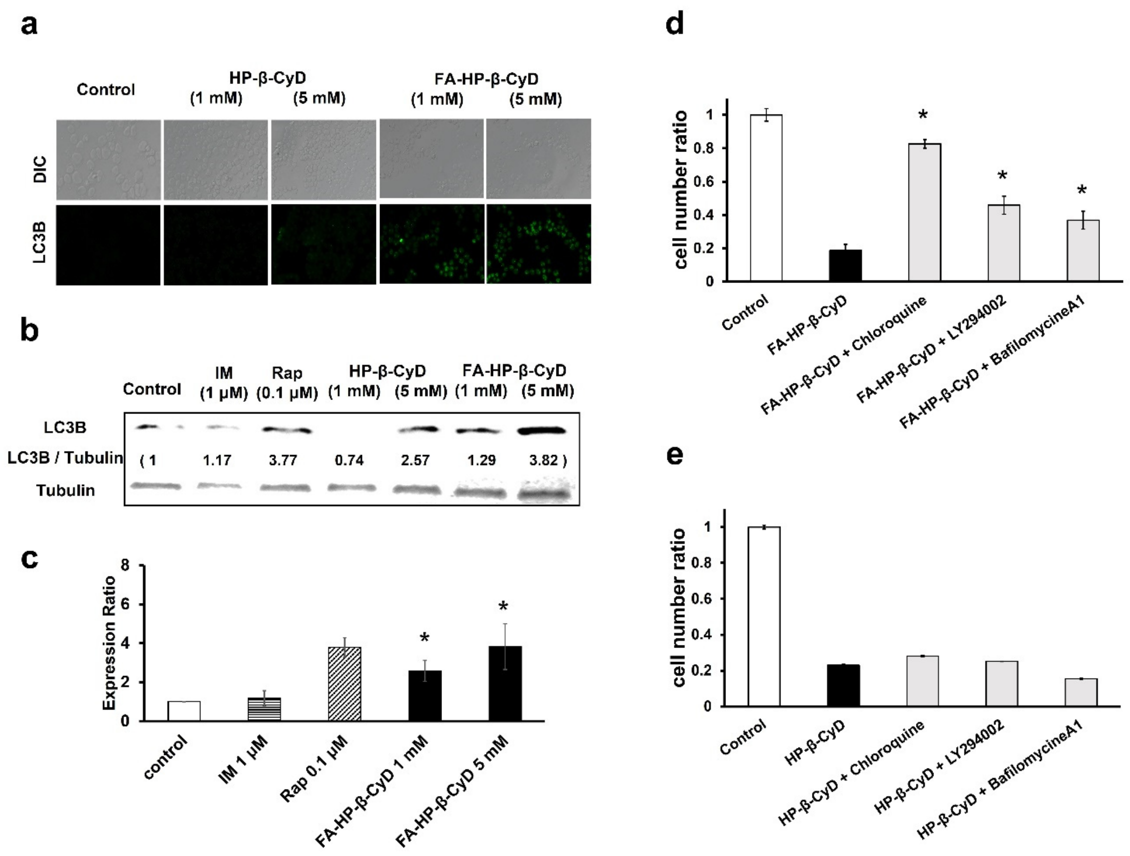

Figure S3. Induction of autophagy by FA-M-β-CyD. (a) Detection of autophagy in CML cells. BV173 cells were treated with FA-HP-β-CyD and HP-β-CyD for 2 h. Then, cells were treated with Cyto-ID for 30 min. Cells were observed by fluorescence microscopy. (b) Effect of HP-β-CyDs on LC3B expression in BV173 cells. Cells were treated with FA-HP-β-CyD, HP-β-CyD, imatinib (IM), and rapamycin (Rap) for 2 h. LC3B protein levels were detected by western blotting. (c) The graph shows the fluorescence intensity of bands. *P < 0.05 compared with control. (d and e) Effects of chloroquine, bafilomycin A1, and LY294002 on the antitumor activity of FA-HP-β-CyD (d) and HP-β-CyD (e) in BV173 cells. *P < 0.05 compared with FA-HP-β-CyD.

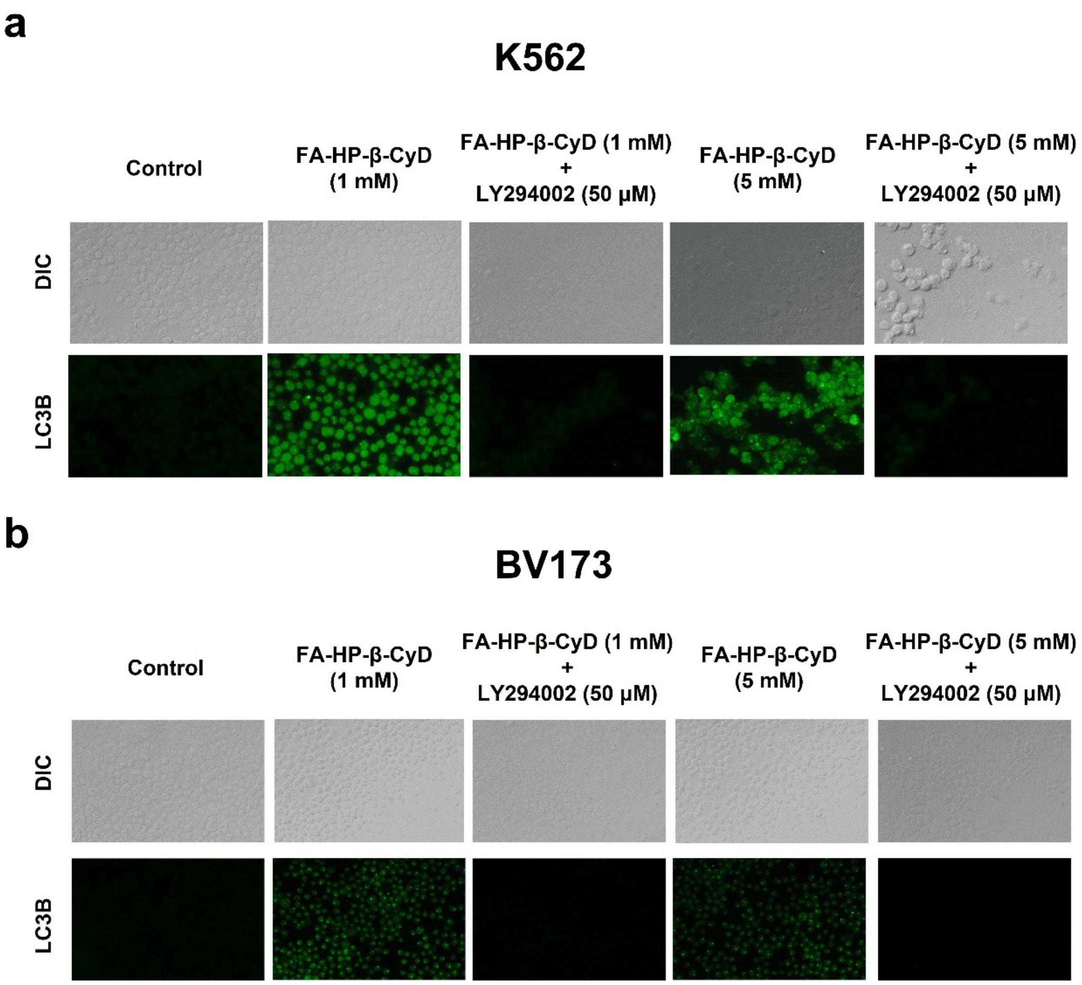

Figure S4. Effect of an autophagy inhibitor on FA-HP-β-CyD. K562 (a) and BV173 cells (b) were incubated with medium containing FA-HP-β-CyD (1 mM, 5 mM) and HP-β-CyD (1 mM, 5 mM) in the absence or presence of LY294002 (50 μM). Cells were stained with LC3B antibody and observed under a fluorescence microscope.

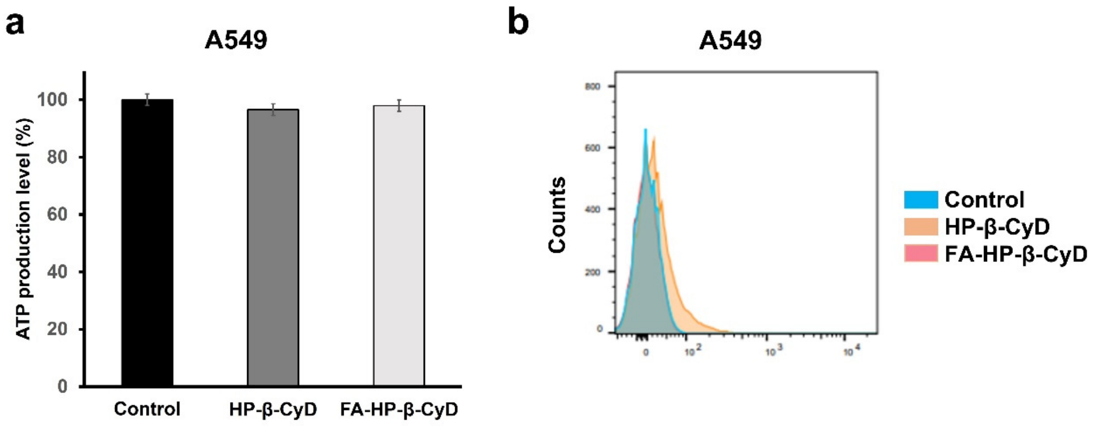

Figure S5. Measurement of intracellular ATP levels and ROS generation in A549 cells. (a) A549 cells were incubated with 1 mM FA-HP-β-CyD and 10 mM HP-β-CyD for 2 h. Then, the cells were treated with ATP detection reagent. Bar graphs represent the mean ± SEM (n=3 per group). (b) A549 cells were incubated with 1 mM FA-HP-β-CyD and 10 mM HP-β-CyD, and then detected by fluorescence cytometry using ROS detection reagents.

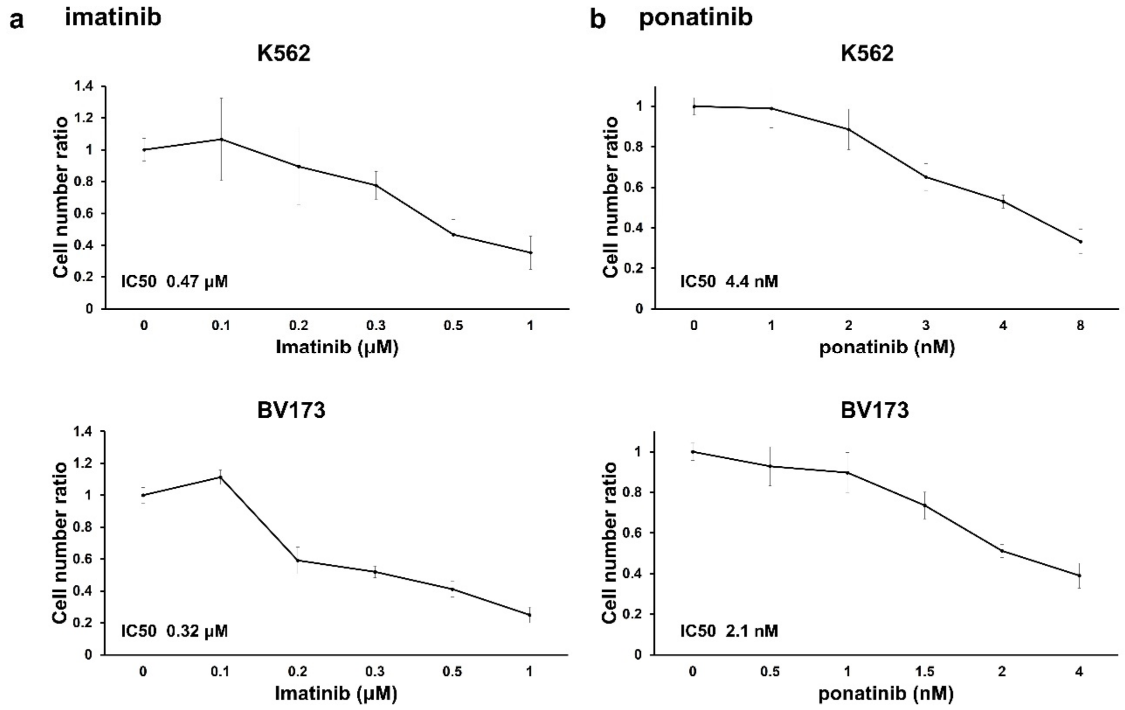

Figure S6. Antitumor activity of imatinib and ponatimib. (a) Cell growth inhibitory effect of imatinib in K562 and BV173 cells. IC_{50} is shown. (b) Cell growth inhibitory effect of ponatinib in K562 and BV173 cells. IC_{50} is shown.

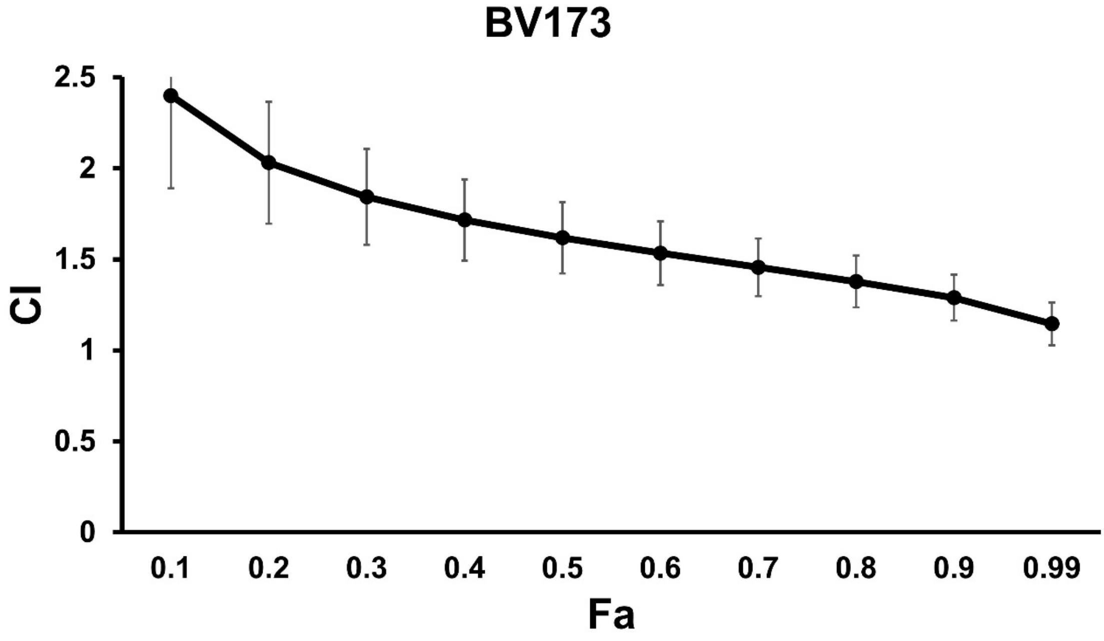

Figure S7. Evaluation of the combined effect of HP-β-CyD and imatinib on BV173 cells. (a) The combination index (CI) was plotted as a function of the fraction affected (Fa), which represents the percentage of growth inhibition (e.g., 0.5 = 50%). Combinations of multiple equipotent drug concentrations were analyzed for synergistic (CI < 1), additive (CI = 1), or antagonistic (CI > 1) effects. Data are presented as the mean ± SD of three independent experiments.

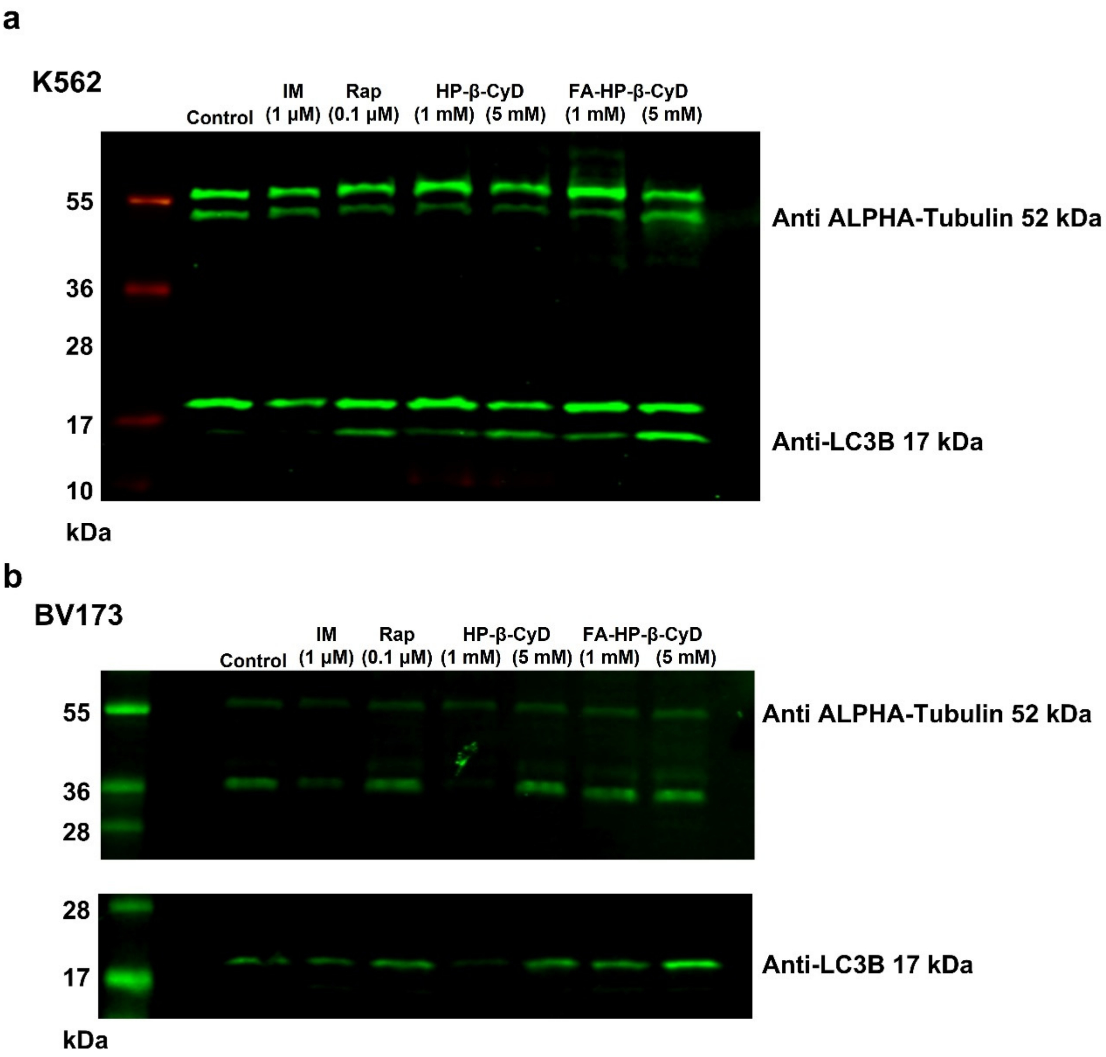

Figure S8. Original western blot figures.
